# Clinical Implication of Corrective Saccades in the Video Head Impulse Test for the Diagnosis of Posterior Inferior Cerebellar Artery Infarction

**DOI:** 10.3389/fneur.2021.605040

**Published:** 2021-02-18

**Authors:** Gi-Sung Nam, Hyun-June Shin, Jin-Ju Kang, Na-Ri Lee, Sun-Young Oh

**Affiliations:** ^1^Department of Otorhinolaryngology – Head and Neck Surgery, Chosun University College of Medicine, Gwangju, South Korea; ^2^Research Institute of Clinical Medicine, Jeonbuk National University Hospital-Biomedical Research Institute, Jeonbuk National University, Jeonju, South Korea; ^3^Department of Neurology, School of Medicine, Jeonbuk National University, Jeonju, South Korea; ^4^Division of Oncology and Hematology, Department of Internal Medicine, School of Medicine, Jeonbuk National University, Jeonju, South Korea

**Keywords:** head impulse test, vestibulo-ocular reflex, posterior inferior cerebellar artery, vertigo, acute vestibular syndrome, corrective saccades, VOR gain

## Abstract

**Objective:** In the present study, we characterized the vestibulo-ocular reflex (VOR) gain and properties of corrective saccades (CS) in patients with posterior inferior cerebellar artery (PICA) stroke and determined the best parameter to differentiate PICA stroke from benign peripheral vestibular neuritis (VN). In particular, we studied CS amplitude and asymmetry in video head impulse tests (vHITs) to discriminate these two less-studied disease conditions.

**Methods:** The vHITs were performed within 1 week from symptom onset in patients with PICA stroke (*n* = 17), patients with VN (*n* = 17), and healthy subjects (HS, *n* = 17).

**Results:** PICA stroke patients had bilaterally reduced VOR gains in the horizontal semicircular canal (HC) and the posterior semicircular canal (PC) compared with HSs. When compared with VN patients, PICA stroke patients showed preserved gains in the HC and anterior semicircular canal (AC) bilaterally (i.e., symmetric VOR gain). Similar to VOR gain, smaller but bilaterally symmetric CS in the HC and AC were observed in PICA stroke patients compared with VN patients; the mean amplitude of CS for the ipsilesional HC was reduced (*p* < 0.001, Mann–Whitney *U*-test), but the mean amplitude of CS for the contralesional HC was increased (*p* < 0.03, Mann–Whitney *U*-test) in PICA stroke compared with VN. The receiver operating characteristic (ROC) curve showed that CS amplitude asymmetry (CSs) and VOR gain asymmetry (Gs) of HC are excellent parameters to distinguish PICA stroke from VN.

**Conclusion:** In the current study, we quantitatively investigated the VOR gain and CS using vHITs for three semicircular canals in PICA stroke and VN patients. In addition to VOR gain, quantitative assessments of CS using vHITs can provide sensitive and objective parameters to distinguish between peripheral and central vestibulopathies.

## Introduction

Acute vestibular syndrome (AVS) is characterized by the presence of acute continuous vertigo, motion intolerance, and gait unsteadiness lasting longer than 24 h (1). Vestibular neuritis (VN) and posterior circulation stroke are the most common causes of AVS in peripheral and central vestibular disorders, respectively (2). The head impulse test (HIT) is a useful tool to evaluate the vestibulo-ocular reflex (VOR) during relatively rapid head rotation and can help to discriminate whether vestibular disorder syndromes are directly involved with the VOR pathway (3). Typically, a pathological HIT demonstrating decreased gain with corrective saccades (CS) is interpreted as a sign of a peripheral vestibulopathy in clinical practice. Recent observations made during quantitative assessments have indicated that central vestibular disorders demonstrate specific patterns of abnormalities (4–8). Patients with lesions involving the vestibular nucleus, nucleus prepositus hypoglossi, or flocculus show reduced gains unilaterally or bilaterally or diffuse cerebellar lesion-induced increased gains with CS or cross-coupled vertical CS. In addition, patients with vestibular nucleus, medial longitudinal fasciculus, or cerebellar lesions show decreased (hypoactive) or increased (hyperactive) VOR gains during vertical HITs (7). In contrast to anterior inferior cerebellar artery (AICA) or lateral medullary strokes, which usually present as dramatic neurologic deficits with hearing loss or brainstem signs, most cases of cerebellar stroke in the posterior inferior cerebellar artery (PICA) present with normal or subtle cerebellar signs (9). Furthermore, in quantitative HIT studies, AICA–PICA strokes with brainstem lesions usually showed bilateral low gain, while patients with pure cerebellar infarction of the PICA territories had gain values similar to those of healthy controls (6). In addition, PICA stroke involving the nodulus and uvula, which have strong connections to the vestibular nuclei (10, 11), may cause spontaneous and/or head-shaking nystagmus similar to VN (i.e., pseudo-VN) (9, 12). Approximately 10% of patients with cerebellar stroke, especially in the medial branch of the PICA, are thought to have isolated vertigo. However, it is not always possible for clinicians to differentiate this from peripheral vertigo in patients with AVS (9). In a prior search coil study, patients with PICA stroke showed bilateral mild gain reductions with small CS and minimal gain asymmetry in the horizontal semicircular canal (HC) plane (4), and the presence of large-amplitude CS with a profound right–left asymmetry indicated a peripheral vestibular lesion (4, 13). In the present study, only cerebellar PICA strokes were included, but AICA or lateral medullary strokes were not included, which is a major limitation of our study. Further vHIT studies should explicitly examine AICA and lateral medullary strokes. Recent reports have evaluated the properties of CS including their latency, frequency, and amplitude as well as the presence of CS in peripheral vestibular disorders; however, these existing studies focused on elucidating HC VOR gain using clinical or quantitative HIT. Relatively few studies have included the vertical canals systematically with quantitative CS, especially in patients with PICA stroke. Herein, VOR gain and CS properties for individual semicircular canals were investigated in a group of AVS patients (PICA stroke and VN), using the video HIT (vHIT) to supplement diagnostic characteristics and determine which parameters might optimally differentiate PICA from VN.

## Materials and Methods

### Subjects

This single-center study included 17 patients with PICA stroke confirmed by magnetic resonance imaging (MRI) and 17 patients with VN who were prospectively enrolled from March to August 2019 at Jeonbuk National University Hospital. Based on data from previous studies, we estimated that the proportion of patients with CS would be 90% in the VN group and 60% in the PICA group. By adopting a power of 0.8 to detect a significant difference (*p* = 0.05, two-sided) and 0.05 alpha and 0.2 beta, 16 patients (32 in total) were required for each study group. The study participants were included based on the presence of AVS symptoms with acute continuous vertigo, nausea or vomiting, and gait disturbance for <7 days after symptom onset. Patients with a previous history of stroke or vestibular disorder were excluded, as well as patients with lateral medullary (*n* = 5) or other brainstem or supratentorial stroke (*n* = 13). Table 1 summarizes patient demographics and neurologic findings. The 17 PICA stroke patients were examined by two neurotologists (S.Y. Oh and H.J. Shin), and the PICA territory lesions were confirmed based on diffusion-weighted MRI. VN patients (*n* = 17) were diagnosed based on history of acute-onset vertigo with the presence of unidirectional spontaneous nystagmus that obeyed Alexander's law, abnormal clinical HIT and caloric paresis, and the absence of neurotologic signs. Eleven VN patients who had multiple vascular risk factors, accompanying headache or prolonged vertigo for 3 days, underwent brain MRI and were confirmed to be stroke free. The six VN patients who showed symptoms and signs of typical peripheral vestibular syndrome on clinical neurotology examination did not undergo a brain MRI and fully recovered after conservative management for several days. In addition, we included 17 healthy subjects (HS) with no neurologic or vestibular disorders or any previous history of stroke or vestibular disorders. There were no statistical differences in age and sex between the groups (Table 1). All subjects underwent neurotologic evaluations including video oculography, vHIT, and pure-tone audiometry.

**Table 1:** Clinical characteristics and neurotologic signs in patients with PICA stroke and VN.

	**PICA stroke (*n* = 17)**	**VN (*n* = 17)**	**HS (*n* = 17)**	***p*-value**
Age	65.8 ± 13.7	56.5 ± 16.0	57.9 ± 8.3	*0.18*^*a*^
Sex (M: F)	9:8	11:6	8:9	*0.48*^*b*^
Lesion (R: L)	9:8	10:7	-	*0.43*^*b*^
Neurotologic signs				
Spontaneous nystagmus	12 (70.6%)	15 (88.2%)	-	*0.40*^*b*^
Direction-changing nystagmus	7 (41.2%)	0	-	* <0.01*^*b*^
Skew deviation	2 (11.8%)	0	-	*0.49*^*b*^
Abnormal clinical HIT	3 (17.7%)	15 (88.2%)	-	* <0.001*^*b*^
Severe gait ataxia^c^	10 (58.8%)	0	-	* <0.001*^*b*^
Acute hearing loss	0	0	0	*-*
Mean time from symptom onset to vHIT	4.6 days	6.7 days	-	*0.71*^*a*^

*PICA, posterior inferior cerebellar artery; VN, vestibular neuritis; HS, healthy subjects; M, male; F, female; R, right; L, left; HIT, head-impulse test*.

a Kruskal–Wallis test;

b Fisher's exact test;

c *patients with severe ataxia who cannot stand unaided*.

PICA stroke was subclassified as medial PICA (PICA_M_) stroke when the lesion involved the nodulus or uvula and lateral PICA (PICA_L_) stroke when the lesion did not affect the nodulus and uvula. The PICA_M_ and PICA_L_ groups consisted of eight patients (five males; 63.0 ± 5.43 years of age) and nine patients (four males; 68.2 ± 12.3 years of age), respectively. Because there is no previous study where the variances and prevalence rates of VOR gain and CS amplitudes in the subgroups of PICA_M_ and PICA_L_ are estimated in a blinded fashion, the sample size for the subgroup analysis could not be recalculated accordingly. However, if we adopt a power of 0.8 to detect a significant difference based on VOR gains of 0.9 and 0.7 in each PICA_M_ and PICA_L_ group, nine patients (18 patients) were required for each subgroup. There were no statistical differences in age or gender ratio between the two groups.

All subjects signed informed consent and received monetary compensation for their participation. This study was approved by the Institutional Review Board at Jeonbuk National University Hospital (no. 2019-04-051).

### Video-Oculography (VOG)

Eye movement and gaze stability were examined using three-dimensional video oculography (3D-VOG, SMI, the Netherlands). Eye movements and the ability to hold a steady gaze were evaluated during attempted fixation on visual targets located centrally or eccentrically (±30° horizontally and ±20° vertically). Spontaneous and gaze-evoked nystagmus, vibration and head-shaking nystagmus, positional tests, and horizontal saccade and smooth pursuit eye movements were evaluated. Digitized data were analyzed using MATLAB® software (MathWorks, Natick, MA, USA).

### vHIT

The vHIT was performed with a videonystagmography system (SLMED, Seoul, Korea). Subjects were positioned at a distance of 1 m from a target located at eye level. To ensure reliability of the examination process, goggles were fastened to the head with an elastic band to minimize slippage. The subjects were seated in a height-adjustable chair which allowed the examiner to adjust the height of each subject's head to an optimal level for the examination. Subjects were instructed to watch the point on the wall 1 m ahead. The examination was conducted by an experienced technician who was blinded to the results of the clinical and MRI findings. The technician manually performed rotation more than 20 times (head rotation 15–20°, duration 150–200 ms, peak velocity >150°/s) on both sides of each plane. Because accurate and consistent angles for vertical semicircular canal head rotations are difficult to obtain in the vHIT, an experienced well-trained technician performed all maneuvers.

After calibrating the eye position, the technician applied a series of horizontal head impulses to the right and left in a random order to measure and record eye movement. Vertical impulses were applied along the right and left vertical canal planes (14). The vHIT parameters used in quantitative analysis were VOR gain, VOR gain asymmetry (Gs), CS amplitude, CS amplitude asymmetry (CSs), and CS latency. The VOR gain of the three semicircular canals was calculated using the ratio of the area under the curve (AUC) for eye velocity area divided by head velocity area, as automatically determined by the device. A normal vHIT result is defined as a gain within two standard deviations (SDs) of the age-matched normal gain reference range without fixation CS. The CS used in the analysis was defined as the occurrence of more than 20% of trials with similar amplitude and latency during all vHIT trials, and results out of range were excluded from the analysis. In addition, to reduce artifacts, peak velocities were maintained above 150°/s, and trace oscillations during head movement, pseudo-saccades (blinks), head-motion artifacts, and other tracking errors were excluded from the analysis (15). We only analyzed the incidence and properties of overt saccades (i.e., cases where a CS occurred after the point when the head velocity crossed zero). The CS amplitude was defined as the area of CS and was calculated using the Engauge Digitizer graphics software program after each trial was independently extracted (16). CS latency was defined from the start of the head impulse to the point when the CS showed peak amplitude. The amplitude and latency of CS were reported in degrees (°) and referred to the area of CS and milliseconds (ms), respectively. The VOR Gs and CSs were determined as the absolute difference obtained by subtracting the value of the ipsilesional side from the contralesional side.

### Data Availability Statement

All the individual participant data that underlie the results reported in this article after deidentification (text, tables, and figures) will be shared.

### Statistical Analysis

All statistical analyses were performed using the Statistical Package for the Social Sciences for Windows version 20.0 (IBM Corp., Armonk, NY, USA). A *p*-value < 0.05 was considered to indicate statistical significance. Quantitative parameters were described by means ± SD. The independent *t*-test, Mann–Whitney *U*-test, one-way analysis of variance (ANOVA), Kruskal–Wallis test, and Fisher's exact test were used to compare parameters in this study. Each evaluation method was selected based on the normality of the sample. Receiver operating characteristic (ROC) curve analysis was performed to identify which parameters were useful to differentiate PICA stroke from peripheral AVS.

## Results

Clinical characteristics and neurotologic signs in patients with PICA stroke and VN are summarized in Table 1. The PICA stroke group had more patients with direction-changing nystagmus, negative clinical HIT findings, and severe ataxia who could not stand unaided compared to the VN group (Table 1).

### VOR Gains in PICA Stroke and VN

**Figure 1:**
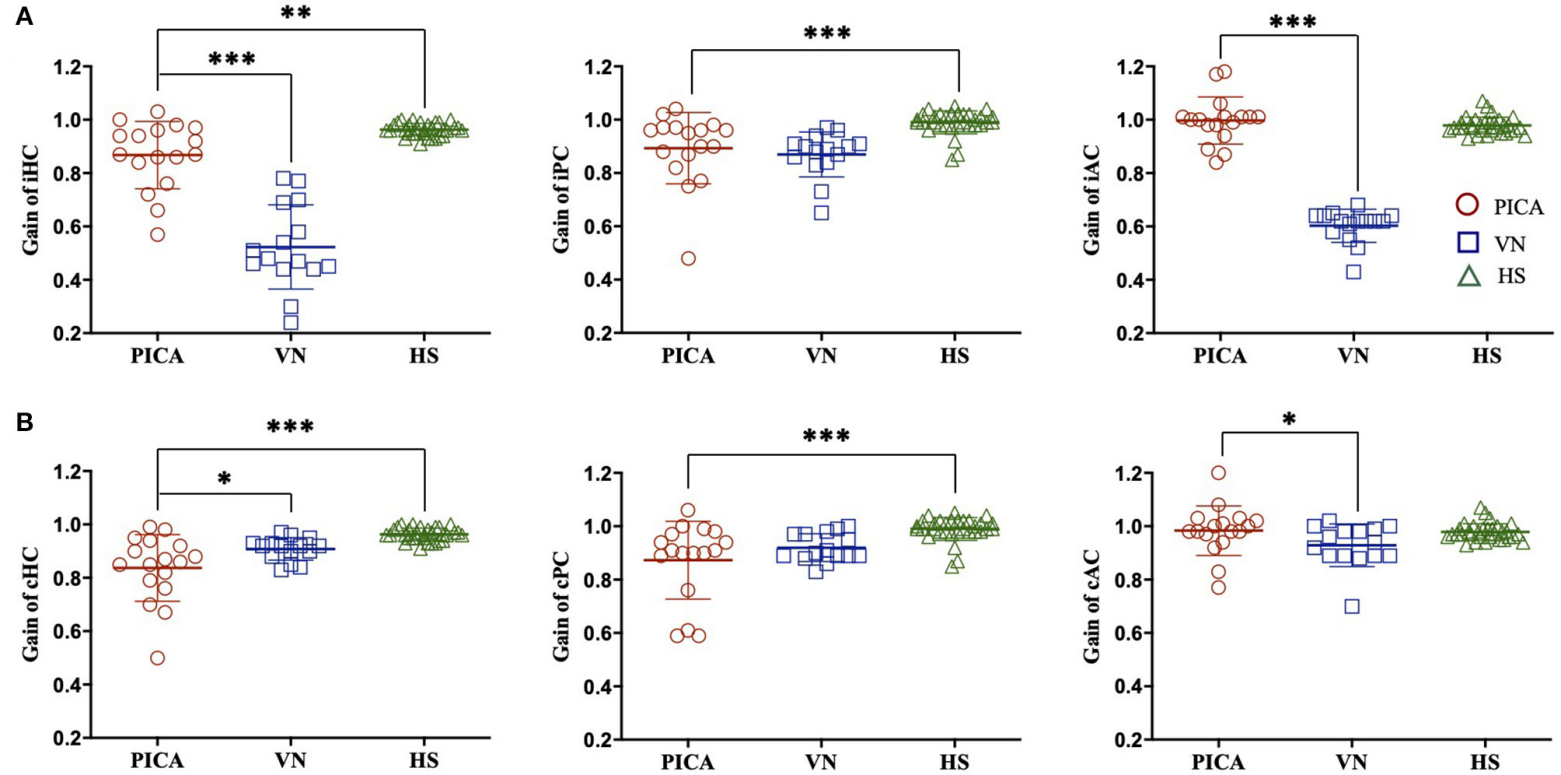
VOR gain of the **(A)** ipsilesional (i) and **(B)** contralesional (c) sides of three semicircular canals in patients with PICA stroke, patients with VN, and HS. Compared with the HS group, the VOR gain for PICA stroke patients was reduced bilaterally in the HC and PC but preserved in the AC. Compared with the VN group, the VOR gain of PICA stroke patients was increased in the iHC, iAC, and cAC. ***p-value < 0.001, **p-value < 0.01, *p-value < 0.05. PICA, posterior inferior cerebellar artery; VN, vestibular neuritis; HS, healthy subjects; VOR, vestibulo-ocular reflex; AC, anterior semicircular canal; HC, horizontal semicircular canal; PC, posterior semicircular canal.

**Figure 2:**
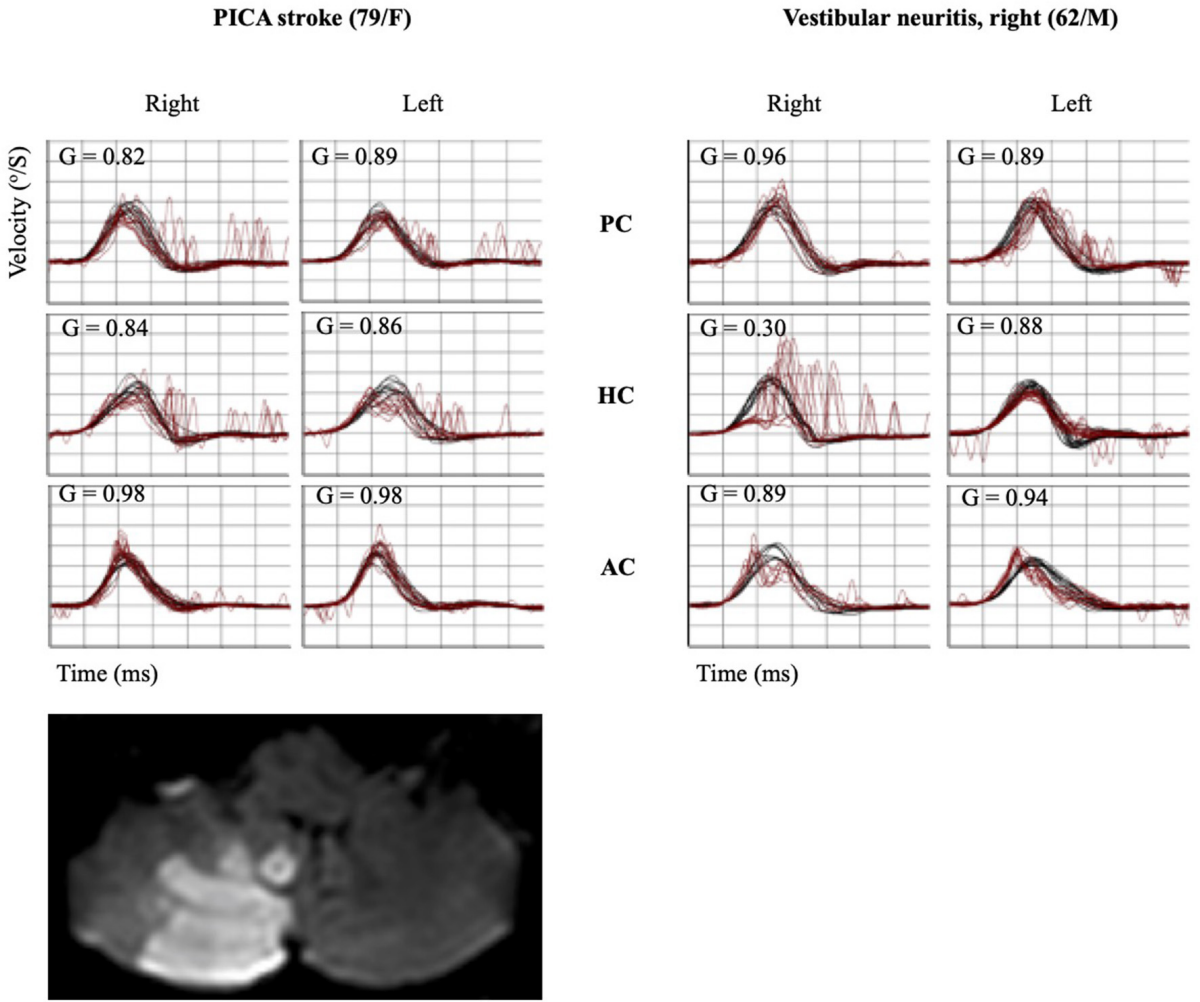
Quantitative HIT recordings and MRI findings of representative PICA stroke and VN subjects. A patient with PICA stroke showed mildly decreased gain bilaterally in the HC and PC with a small CS bilaterally in the same canals. Decreased VOR gain with corrective catch-up saccades was observed in the ipsilesional HC and AC using the vHIT in a patient with VN. HIT, head impulse test; MRI, magnetic resonance imaging; PICA, posterior inferior cerebellar artery; VN, vestibular neuritis; AC, anterior semicircular canal; HC, horizontal semicircular canal; PC, posterior semicircular canal; CS, corrective saccade; VOR, vestibulo-ocular reflex; vHIT, video head impulse test.

The difference in VOR gain among subjects with PICA stroke, VN, and HS is detailed in Figure 1 and Table 2. The VOR gains were symmetric (1.8 ± 1%) and just below 1 [mean VOR gain ± SD: 0.96 ± 0.02, 0.99 ± 0.04, and 0.98 ± 0.03 for the HC, posterior semicircular canal (PC), and anterior semicircular canal (AC), respectively] in the HS group (green triangles in Figure 1). In the VN group (*n* = 17), gains were smaller, predominantly in the ipsilesional HC and AC, and were reduced by ~20–35% contralesionally, leading to marked asymmetry (Table 2, blue squares in Figures 1, 2). In contrast with the HS, patients with PICA stroke showed mildly deficient HC and PC VOR gains bilaterally (0.87 ± 0.13 and 0.89 ± 0.13, *p* = 0.003 and *p* < 0.001 for the ipsilesional HC and PC, respectively, Mann–Whitney *U*-test; 0.84 ± 0.13 and 0.87 ± 0.15, *p* < 0.001 and *p* < 0.001 for the contralesional HC and PC, respectively, Mann–Whitney *U*-test; Table 2, red circles in Figures 1, 2). However, the AC gain was preserved bilaterally (1.00 ± 0.09 and 0.98 ± 0.09, *p* = 0.10 and *p* = 0.27 for ipsilesional AC and contralesional AC, respectively, Mann–Whitney *U*-test). When compared with the VN group, subjects in the PICA stroke group showed relatively preserved gains in the HC and AC bilaterally (i.e., symmetric gain on both sides), resulting in differences in Gs between PICA stroke and VN patients during the HC and AC examinations (*p* < 0.001, Mann–Whitney *U*-test; Table 2).

**Table 2:** VOR gain of quantitative HIT in patients with PICA stroke and VN.

**Gain, mean ± 2SD**	**PICA (*****n*** **=** **17)**		**VN (*****n*** **=** **17)**		**HS (*n* = 17)**	***p*****-value (PICA vs. VN)**		***p-*****value (PICA vs. HS)**	
**(95% CI)**	**i**	**c**	**i**	**c**		**i**	**c**	**i**	**c**
HC	0.87 ± 0.13 (0.80–0.93)	0.84 ± 0.13 (0.77–0.90)	0.52 ± 0.16 (0.44–0.61)	0.91 ± 0.04 (0.88–0.93)	0.96 ± 0.02 (0.95–0.97)	* <0.001^*a*^*	*0.045^*a*^*	*0.007^*a*^*	* <0.001^*a*^*
PC	0.89 ± 0.13 (0.82–0.96)	0.87 ± 0.15 (0.80–0.95)	0.87 ± 0.09 (0.82–0.92)	0.92 ± 0.05 (0.89–0.95)	0.99 ± 0.04 (0.98–1.00)	*1.00^*b*^*	0.97^*b*^	* <0.001^*a*^*	* <0.001^*b*^*
AC	1.00 ± 0.99 (0.95–1.04)	0.98 ± 0.09 (0.94–1.03)	0.60 ± 0.06 (0.57–0.64)	0.93 ± 0.08 (0.88–0.97)	0.98 ± 0.03 (0.97–0.99)	* <0.001^*b*^*	*0.049^*b*^*	*0.098^*b*^*	*0.268^*a*^*
Abnormal gain, *n* (%)^e^									
HC	9 (52.9%)	12 (70.6%)	17 (100%)	4 (23.5%)	2 (5.9%)	*0.003^*c*^*	*0.004^*c*^*	* <0.001^*c*^*	* <0.001^*c*^*
PC	8 (48.1%)	10 (58.8%)	8 (47.1%)	10 (58.8%)	2 (5.9%)	*0.98^*c*^*	*0.95^*c*^*	* <0.001^*c*^*	* <0.001^*c*^*
AC	3 (17.6%)	2 (11.7%)	12 (70.6%)	7 (41.2%)	0 (0%)	*0.02^*c*^*	*0.11^*c*^*	*0.033^*c*^*	*0.107^*c*^*
Gs^d^ (95% CI)									
HC	0.08 ± 0.08 (0.04–0.12)		0.38 ± 0.17 (0.29–0.48)		0.02 ± 0.02 (0.01–0.03)	* <0.001^*b*^*		*0.002^*b*^*	
PC	0.06 ± 0.05 (0.03–0.09)		0.07 ± 0.06 (0.03–0.10)		0.03 ± 0.04 (0.01–0.05)	*0.71^*b*^*		*0.045^*b*^*	
AC	0.05 ± 0.06 (0.02–0.08)		0.33 ± 0.04 (0.29–0.36)		0.04 ± 0.03 (0.02–0.05)	* <0.001^*b*^*		*0.929^*b*^*	
Abnormal gain asymmetry, *n* (%)^e^									
HC	9 (52.9%)		17 (100%)		1 (5.9%)	*0.003^*c*^*		*0.003^*c*^*	
PC	4 (23.5%)		3 (20.0%)		1 (5.9%)	*0.81^*c*^*		*0.335^*c*^*	
AC	2 (11.8%)		5 (33.3%)		1 (5.9%)	*0.21^*c*^*		*1.000^*c*^*	

*PICA, posterior inferior cerebellar artery; VN, vestibular neuritis; HS, healthy subjects; SD, standard deviation; CI, confidence interval; HC, horizontal canal; PC, posterior canal; AC, anterior canal; Gs, gain asymmetry; i, ipsilesional; c, contralesional*.

a Independent t-test;

b Mann–Whitney U test;

c Fisher's exact test;

d gain asymmetry was defined as the absolute difference between ipsilesional and contralesional gains;

e *abnormal gain and abnormal gain asymmetry were defined as −2 SDs below the mean and as above mean +2 SDs of 95% of normal subjects incorporated, respectively. The value in parentheses below the mean ± SD represents the 95% CI*.

**Figure 3:**
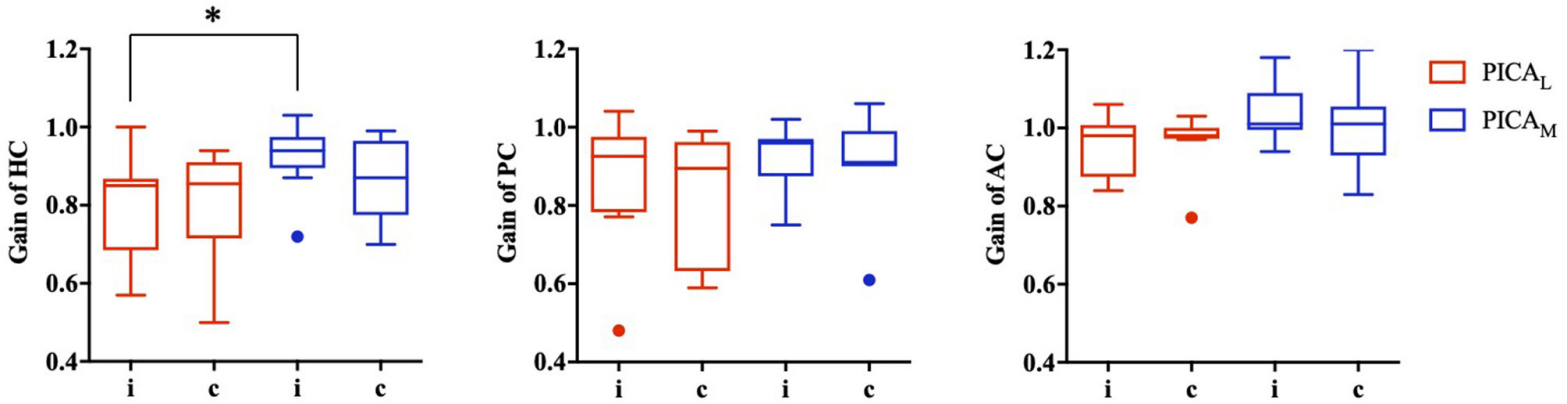
VOR gain for medial (PICA_M_) and lateral (PICA_L_) PICA stroke based on the involvement or non-involvement (PICA_L_) of the nodulus or uvula (PICA_M_). The VOR gain for PICA_M_ tended to increase more than PICA_L_ in all three semicircular canals; however, statistical significance was observed only in the ipsilesional horizontal semicircular canal (*p-value < 0.05, Mann–Whitney *U*-test).

The data for patients with PICA stroke were subanalyzed based on the involvement of the nodulus and uvula (PICA_M_ group: *n* = 8, mean age: 63.0 ± 15.4 years) or non-involvement (PICA_L_ group: *n* = 9, mean age: 68.2 ± 12.4 years). The VOR gains for the PICA_M_ group generally increased more than those for the PICA_L_ group on both sides in all three semicircular canals; however, statistical significance was observed only in the ipsilesional HC (*p* = 0.027, Mann–Whitney *U*-test; Figure 3).

### Characteristics of CS in PICA Stroke and VN

**Figure 4:**
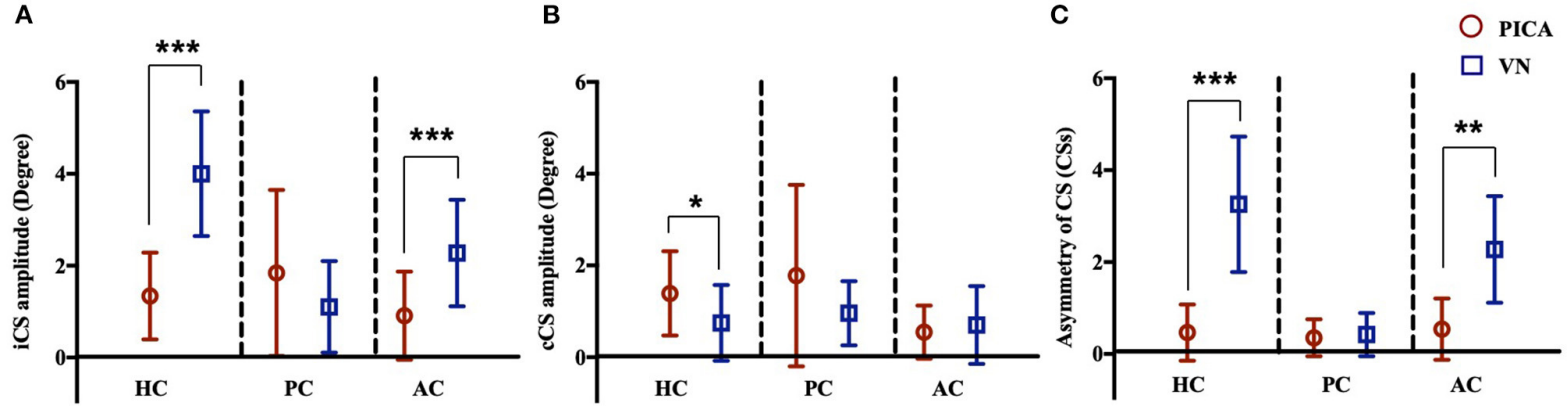
CS amplitude of the **(A)** ipsilesional (i) and **(B)** contralesional (c) sides of three semicircular canals in patients with PICA stroke and VN. In patients with PICA stroke, the CS amplitudes for the iHC and iAC were reduced compared with those of VN patients (*p* < 0.001 and *p* < 0.001 in the iHC and iAC, respectively, Mann–Whitney *U*-test) **(A)**. The CS amplitude of the cHC was increased in PICA stroke compared with VN patients (mean CS amplitude: 1.39 ± 0.92 in PICA vs. 0.75 ± 0.82 in VN; *p* = 0.03, Mann–Whitney *U*-test) **(B)**. The asymmetry of CSs in PICA stroke was significantly decreased in the HC (mean CSs of HC, 3.26 in VN vs. 0.47 in PICA, *p* < 0.001, Mann–Whitney *U*-test) and AC compared with VN patients **(C)**. ***p < 0.001, **p < 0.01, *p < 0.05. AC, anterior semicircular canal; HC, horizontal semicircular canal; PC, horizontal semicircular canal.

The incidence (%) and amplitude (°) of CS and CSs in patients with PICA and VN are summarized in Table 3 and Figure 4. In the HS group, saccades were small and symmetric and were observed in ~10–18% of cases for each semicircular canal, which is consistent with other reports (17). VN patients showed frequent and increased CS mean amplitude during the ipsilesional HC and AC examinations. Compared with VN, patients with PICA stroke usually had smaller but bilateral CS, with frequent contralesional CS on HC and PC examinations (Figure 2 and Table 3). In PICA stroke, bilateral CS was detected (82% in HC, 94% in PC, and 53% in AC) during 20 trials. The degree of CS was negatively correlated with VOR gain on the ipsilesional side (*r* = −0.79 in ipsilesional HC, *r* = −0.87 in ipsilesional PC, and *r* = −0.63 in ipsilesional AC) in PICA stroke patients. Similar to the VOR gain, differences in CS amplitude between PICA stroke and VN patients were apparent in the ipsilesional HC and AC (*p* < 0.001 and *p* < 0.001 for ipsilesional HC and AC, respectively, Mann–Whitney *U*-test; Figure 4A and Table 3). CS amplitude in the ipsilesional HC was markedly different between the PICA and VN groups and was the most valuable parameter for differentiating the two conditions. The mean CS amplitude for contralesional HC was increased in PICA stroke patients compared with VN patients (Figure 4B). Similar to Gs, the robust asymmetry of CSs in the HC and AC was observed in patients with VN (mean CSs of HC: 3.26 in VN vs. 0.47 in PICA; *p* < 0.001, Mann–Whitney *U*-test; Table 3 and Figure 4C). Statistical differences were not observed in CS latencies between the PICA and VN patients; however, CS latencies in the PICA group were relatively decreased compared with HS in the HC and PC (Table 3).

**Table 3:** Properties and incidence of CS in each semicircular canal based on group.

**CS incidence per trial (%)**	**PICA (*****n*** **=** **17)**		**VN (*****n*** **=** **17)**		**HS (*n* = 17)**	***p*****-value (PICA vs. VN)**		***p*****-value (PICA vs. HS)**	
	**i**	**c**	**i**	**c**		**i**	**c**	**i**	**c**
HC	63.3%	70.6%	92.6%	23.2%	18.2%	* <0.001^*a*^*	* <0.001^*a*^*	* <0.001^*a*^*	* <0.001^*a*^*
PC	68.2%	63.8%	52.7%	49.7%	9.4%	* <0.001^*a*^*	* <0.001^*a*^*	* <0.001^*a*^*	* <0.001^*a*^*
AC	27.7%	21.6%	61.1%	23.9%	*5.8%*	* <0.001^*a*^*	*0.54^*a*^*	* <0.001^*a*^*	* <0.001^*a*^*
CS amplitude°, mean ± SD (95% CI)									
HC	1.34 ± 0.94 (0.86–1.83)	1.39 ± 0.92 (0.92–1.86)	4.00 ± 1.36 (3.25–4.75)	0.75 ± 0.82 (0.29–1.20)	0.48 ± 0.29 (0.38–0.58)	* <0.001^*b*^*	*0.03^*b*^*	* <0.001^*b*^*	* <0.001^*b*^*
PC	1.84 ± 1.81 (0.91–2.77)	1.78 ± 1.98 (0.76–2.80)	1.10 ± 1.00 (0.55–1.66)	0.96 ± 0.70 (0.57–1.35)	0.27 ± 0.33 (0.15–0.38)	*0.22^*b*^*	*0.37^*b*^*	* <0.001^*b*^*	* <0.001^*b*^*
AC	0.91 ± 0.96 (0.42–1.41)	0.55 ± 0.58 (0.25–0.84)	2.28 ± 1.16 (1.63–2.92)	0.70 ± 0.85 (0.23–1.17)	0.25 ± 0.32 *(0.13 ± 0.36)*	* <0.001^*b*^*	*0.74^*b*^*	*0.003^*b*^*	*0.082^*b*^*
CSs^d^ (95% CI)									
HC	0.47 ± 0.61 (0.15–0.78)		3.26 ± 1.47 (2.44–4.07)		0.25 ± 0.32 (0.09–0.41)	* <0.001^*b*^*		*0.081^*b*^*	
PC	0.35 ± 0.40 (0.15–0.56)		0.42 ± 0.47 (0.16–0.68)		0.21 ± 0.25 (0.09–0.33)	*0.85^*b*^*		*0.146^*b*^*	
AC	0.54 ± 0.67 (0.20–0.89)		1.62 ± 0.85 (1.15–2.10)		0.33 ± 0.36 (0.16–0.51)	*0.001^*b*^*		*0.363^*b*^*	
Latency of CS (msec) (95% CI)									
HC	348.5 ± 54.1 (321.1–375.9)	360.3 ± 62.2 (329.9–390.8)	336.1 ± 43.4 (314.1–358.0)	350.0 ± 87.9 (295.5–404.5)	405.3 ± 62.4 (381.8–428.9)	*0.49^*b*^*	*0.73^*b*^*	*0.005^*b*^*	*0.047^*b*^*
PC	374.8 ± 84.5 (333.4–416.2)	368.4 ± 85.2 (326.7–410.2)	338.4 ± 64.9 (299.9–363.9)	326.7 ± 63.1 (289.4–363.9)	431.7 ± 57.2 (404.6–458.9)	*0.24^*b*^*	*0.18^*b*^*	*0.029^*b*^*	*0.019^*b*^*
AC	404.1 ± 95.3 (352.3–455.9)	415.8 ± 73.2 (370.4–461.2)	374.2 ± 75.7 (334.5–413.8)	366.1 ± 84.9 (303.0–429.02)	418.0 ± 40.1 (397.0–439.0)	*0.37^*b*^*	*0.22^*b*^*	*0.544^*b*^*	*0.585^*b*^*
No. of subjects with bilateral CS									
HC	14 (82.4%)		12 (70.6%)		8 (47.1%)	*0.688^*c*^*		*0.031*	
PC	16 (94.1%)		12 (70.6%)		6 (35.3%)	*0.175^*c*^*		* <0.001^*c*^*	
AC	9 (52.9%)		8 (47.1%)		*6 (35.3%)*	*1.000^*c*^*		*0.300^*c*^*	

*PICA, posterior inferior cerebellar artery; VN, vestibular neuritis; HS, healthy subjects; HC, horizontal canal; PC, posterior canal; AC, anterior canal; CS, corrective saccade; SD, standard deviation; CI, confidence interval; i, ipsilesional; c, contralesional*.

a *chi-square test*;

b *Mann–Whitney U test*;

c *Fisher's exact test*;

d *CS asymmetry (CSs) was defined as the absolute difference between ipsilesional and contralesional CS amplitudes. The value in parentheses below the mean ± SD represents the 95% CI*.

### Optimal Cutoff Value of Parameters for Distinguishing PICA From VN

**Figure 5:**
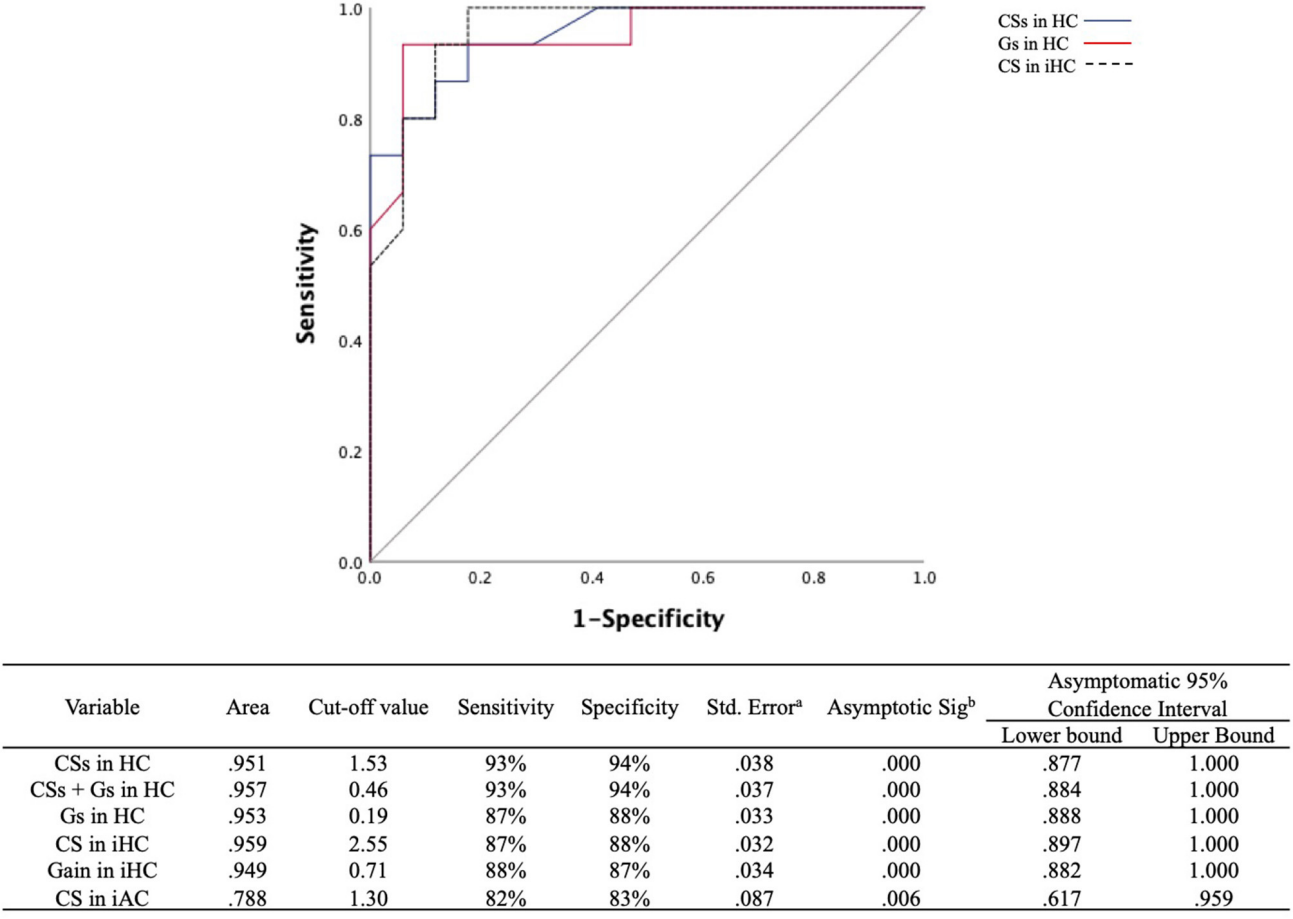
ROC curves for the CSs and Gs in the HC and CS amplitudes in the ipsilesional HC to predict differentiation of PICA stroke from VN. The optimal cutoff value for CSs in the HC provides the best sensitivity (93%) and specificity (94%). The cutoff value for asymmetry of the VOR gain (Gs) in the HC was slightly less sensitive (87%) and specific (88%) than that of CSs in the HC. The gray line indicates a hypothetically useless diagnostic test. ROC, receiver operating characteristic; CSs, asymmetry of corrective saccade; HC, horizontal semicircular canal; Gs, gain asymmetry; AC, anterior semicircular canal. ^a^Under the nonparametric assumption. ^b^Null hypothesis: true area = 0.5.

The ROC curves were plotted to discern which parameters were valuable for discriminating PICA stroke from VN, including ipsilesional and contralesional VOR gains, CS amplitude, Gs, and CSs for all three semicircular canals (Figure 5). We found that CS amplitude of the ipsilesional HC (AUC: 0.959), Gs and CSs of the HC (AUC: 0.953 and 0.951, respectively), and VOR gain of the ipsilesional HC (AUC: 0.949) are robust examples. PICA stroke could be diagnosed with a sensitivity of 87% and specificity of 88% when the CS amplitude of the ipsilesional HC was below 2.55 and with a sensitivity of 88% and specificity of 87% when the VOR gain of the ipsilesional HC was above 0.71. The value for AC showed 82% sensitivity and 83% specificity with a cutoff value of 1.30. Both the ROC curves for Gs and CSs of HC also showed excellent results such as absolute VOR and CS amplitude values; the optimal cutoff value for the Gs of HC above 0.19 showed 87% sensitivity and 88% specificity, and the optimal cutoff value of the CSs for HC was 1.53, providing the best sensitivity (93%) and specificity (94%) outcome (Figure 5). When the CSs and Gs values were combined, the optimal cutoff value for HC above 0.46 showed 93% sensitivity and 94% specificity.

## Discussion

The VOR gain and CS characteristics of HC and vertical semicircular canals were systematically investigated using vHIT in prospectively recruited patients with PICA stroke and VN. The results of the present study were similar to several observations in a prior search coil study which also revealed bilateral mild gain reduction with small CS in the HC plane (4). However, we used vHIT in the present study, as it is clinically more practical than search coil studies, and we also included the vertical canals. In a recent study using vHIT to separate VN from posterior circulation stroke, which included AICA and brainstem strokes as well as PICA strokes, differences were observed in the mean HC VOR gains, CS prevalence, and cumulative CS amplitudes between groups (18). In the current study, the following distinctive vHIT features of PICA stroke compared with VN were observed: (1) preserved VOR gain and smaller CS amplitude in the ipsilesional HC, (2) minimal VOR Gs in the HC, and (3) bilateral and symmetric small CS amplitudes in the HC (Figure 5).

The VOR gains in PICA stroke patients were mildly decreased bilaterally in the ipsilesional and contralesional HC and PC, but not the AC, when compared with HS. When compared with the VN group, VOR gains were significantly preserved in the ipsilesional and contralesional HC and AC (Figure 1 and Table 2). Head impulse with high acceleration saturated the VOR from ipsilateral canal excitation and contralateral canal inhibition. The VOR is better driven by ipsilateral canal excitation than contralateral canal inhibition, especially at higher head acceleration speeds (13). Bilateral gain reductions possibly reflect contributions to the VOR of vestibular commissural inhibition by the contralesional semicircular canals. Unlike some studies showing that cerebellar lesions had no significant effect on vertical HITs (19, 20), our findings revealed that PICA stroke patients had decreased VOR gain in the PC and HC than in the AC bilaterally compared with the HS group. Although PC signals are mainly mediated by the lateral and medial vestibular nuclei, the AC signals are conveyed to the superior vestibular nucleus as well as the lateral and medial vestibular nuclei (21). In addition to the MLF (AC and PC), the brachium conjunctivum pathways are used to carry transmissions from the AC signals to the ocular motor nuclei (22). This partial sparing of the VOR pathway, especially in the AC compared with the PC and HC in PICA stroke, can be explained by the synaptic diversity of the signal transfer process, selective vulnerability of the medial vestibular nucleus (23) or the results of a relatively conserved extra-MLF pathway such as the brachium conjunctivum pathway. The data in the present study also indicate a statistically significant increase in VOR gain in the ipsilesional HC in the PICA_M_ group compared with the PICA_L_ group. The PICA supplies the dorsal vermis, nodulus, and uvula, which are key structures of the vestibulocerebellum. Furthermore, the structures adapt information from the semicircular canals and otoliths to estimate the gravity direction and generate translational VOR using convergent input (24). Reportedly, floccular Purkinje cells affect VOR gain very slightly, but adaptive changes in the VOR are abolished during bilateral destruction of the flocculus (25). In contrast, the ablation of the nodulus and uvula caused a VOR gain increase but only had a minimal effect on the adaptability of the VOR (25). In the group with a medial PICA stroke involving the nodulus and uvula (PICA_M_), patients showed enhanced VOR gain for ipsilesional HC during vHIT (26).

Regarding CS, the bilateral symmetric presence of small-amplitude (1–2°) CS that show mildly reduced amplitude compared with VN is a hallmark of PICA stroke (Table 3). In the present study, considerable attention was given to CS amplitude and asymmetry in vHIT to discriminate PICA stroke from VN, which has been less studied (4). CS analysis is necessary because the quantitative assessment of VOR gain alone does not provide additional information when compared with bedside HIT for differentiation (6). Recently, various indicators, such as maximum velocity, latency, and/or the frequency and presence of CS have been studied for peripheral and central vestibulopathies (17, 18, 27, 28). The velocity or presence of CS was used in the majority of studies (18); however, we have used the average CS value (degree) of each trial, which better correlates with the gain reduction in physiologic aspects. The CS amplitude was defined as the AUC instead of the maximal amplitude of CS, and the degree of CS was calculated, which represented both temporal and spatial concepts in a manner similar to the VOR gain measurement. Because the degree of saccades usually correlates with the VOR gain on the ipsilesional side, the compensatory mechanism in response to decreased VOR gain has been suggested as a mechanism of CS. In contrast to VN patients who showed large CS on the ipsilesional side, PICA stroke patients characteristically had small, frequent CS on the ipsilesional and contralesional sides (i.e., bilateral symmetric CS; Table 3). The asymmetry of CS amplitude in the ipsilesional and contralesional sides (CSs) can be used to differentiate PICA stroke and VN (Figure 5). The difference in CS amplitude between PICA stroke and VN may be due to the severity of gain reduction or by partial disruption of projections from the nodulus to the vestibular nucleus. The nodulus might be implicated because it modulates saccades. Because a dorsal vermis lesion causes ipsilesional hypometria (29), another explanation is that CS might represent refixating eye movements in the presence of saccadic undershooting in PICA stroke patients (29, 30).

Notably, in the present study, the CS amplitude of ipsilesional HC was the best measure for differentiating PICA stoke from VN (AUC: 0.959; Figure 5). However, PICA stroke shows relatively symmetric and mild VOR dysfunction compared to VN, and it can be difficult to determine PICA lesion laterality prior to MRI. Therefore, CSs and VOR Gs are more valuable parameters than the absolute values of CS and VOR gain for distinguishing between these two conditions. ROC analysis using a cutoff value of CSs in HC of 1.53 or less (AUC: 0.951) improved diagnostic accuracy; 94.1% of PICA strokes were correctly diagnosed, and ~93.3% of VNs were detected, which are better outcomes than the 87% sensitivity and 88% specificity associated with the asymmetry of VOR gain (Gs) in HC. However, the combination of the CSs and Gs cutoff values did not improve the power of diagnostic certainty (Figure 5). In a previous search coil study, the cumulative amplitude of CS during horizontal HIT was smaller compared with posterior circulation strokes including AICA and SCA strokes as well as PICA strokes (4). The authors also concluded that when using additional saccade analyses, pontine-cerebellar stroke could be detected with a sensitivity of 94–97% and a specificity of 90–100% (4). Another recent study with vHIT revealed when VOR gain and saccade prevalence combined, separated VN from posterior circulation stroke with a sensitivity and specificity of 90.9% (18). Previous and current findings indicate that although HC analysis for VOR gain and CS parameters is valuable, analysis of the vertical canals, especially the PC, is not adequate. Testing all three semicircular canals is substantially more time-consuming than testing only the HC, and vertical canal testing is much more technically demanding than HC testing. Because testing vertical canals does not yield additional information compared with only testing the HC, we suggest that testing the HC and analyzing VOR gain and CS properties may be sufficient from a practical standpoint. Furthermore, if artifacts could be individually managed carefully before analysis, then vHIT is comparable to a search coil study and can provide highly sensitive and specific measures for differentiating PICA stroke from VN.

However, the sensitivity, specificity, or predictive values of the present study findings in PICA stroke should be validated in clinical practice. The results have practical implications. In addition to VOR gain, quantitative assessments of CS during the vHIT can provide objective parameters and offer additional power to differentiate between peripheral and central vestibulopathies such as cerebellar PICA stroke. When performing vHIT, clinicians should compare the difference in CS gain on both sides as well as VOR gain and gain asymmetry.

## Data Availability Statement

The original contributions presented in the study are included in the article/supplementary materials, further inquiries can be directed to the corresponding author.

## Ethics Statement

The studies involving human participants were reviewed and approved by the Institutional Review Board at Jeonbuk National University Hospital (no. 2019-04-051). The patients/participants provided their written informed consent to participate in this study.

## Author Contributions

S-YO, G-SN, H-JS, and J-JK contributed to the design and implementation of the research. H-JS and J-JK contributed to the analysis of the results. S-YO, G-SN, and N-RL contributed to the writing of the manuscript. All authors contributed to the article and approved the submitted version.

## Conflict of Interest

The authors declare that the research was conducted in the absence of any commercial or financial relationships that could be construed as a potential conflict of interest.
